# Gain enhancement of perovskite nanosheets by a patterned waveguide: excitation and temperature dependence of gain saturation

**DOI:** 10.1038/s41377-023-01313-0

**Published:** 2023-11-24

**Authors:** Inhong Kim, Ga Eul Choi, Ming Mei, Min Woo Kim, Minju Kim, Young Woo Kwon, Tae-In Jeong, Seungchul Kim, Suck Won Hong, Kwangseuk Kyhm, Robert A. Taylor

**Affiliations:** 1 Department of Opto & Cogno Mechatronics Engineering, RCDAMP, Pusan National University, Busan, 46241 Republic of Korea; 2 Department of Nano-Fusion Technology, Pusan National University, Busan, 46241 Republic of Korea; 3 Clarendon Laboratory, Department of Physics, University of Oxford, Oxford, OX1 3PU UK

**Keywords:** Fluorescence spectroscopy, Nanoparticles, Micro-optics

## Abstract

Optical gain enhancement of two-dimensional CsPbBr_3_ nanosheets was studied when the amplified spontaneous emission is guided by a patterned structure of polyurethane-acrylate. Given the uncertainties and pitfalls in retrieving a gain coefficient from the variable stripe length method, a gain contour $g(\hslash \omega ,x)$ was obtained in the plane of spectrum energy (ℏ*ω*) and stripe length (*x*), whereby an average gain was obtained, and gain saturation was analysed. Excitation and temperature dependence of the gain contour show that the waveguide enhances both gain and thermal stability due to the increased optical confinement and heat dissipation, and the gain origins were attributed to the two-dimensional excitons and the localized states.

## Abstract (web summary)

Optical gain of perovskite nanosheets becomes increased when a micro-patterned structure is utilized, where gain saturation is analyzed in the plane of spectrum energy and stripe length.

## Graphical abstract

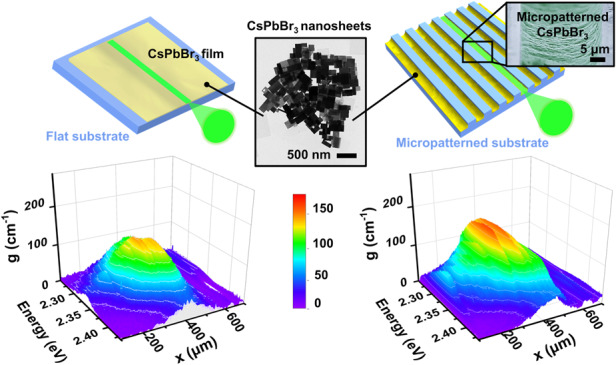

## Introduction

Metal halide perovskites (MHPs) show many outstanding features such as low-cost solution processability, precise bandgap tunability over the entire visible spectral range, and high quantum yield^1,2^, and these advantages can be used in a broad range of applications such as solar cells^3,4^, light-emitting diodes^5,6^, lasers^7–13^, and photodetectors^14,15^. Although organic-inorganic perovskites were studied a great deal, all inorganic perovskites were found to be superior in terms of thermal stability and moisture sensitivity^16,17^.

In particular, MHPs are considered a promising laser medium. In the case of organic-inorganic perovskites such as MAPbI_3_ film, it was reported that a narrow amplified spontaneous emission (ASE) feature emerges near the threshold (~46 µJcm^−2^), and the gain was observed to be 200 cm^−1^ at room temperature under excitation fluence of 100 µJcm^−2^ (see ref. ^18^). On the other hand, all inorganic perovskite CsPbBr_3_ film is known to have a large number of defects in its polycrystalline morphology. Recently, this obstacle was resolved by a large scale single-crystal film of CsPbBr_3_, where a low threshold (∼24 µJcm^−2^) was observed, and the gain was measured to be up to 1369 ± 101 cm^−1^ with an excitation fluence of ∼720 µJcm^−2^ ^10^. CsPbBr_3_ film with small (~200 nm) grains was also seen to exhibit a large gain coefficient (~10^4 ^ cm^−1^) when the excited carrier density is similar to the Mott density (~10^19 ^ cm^−3^)^19^.

In CsPbBr_3_ quantum dots (QDs), the Auger process becomes dominant due to the strong confinement, and the excess energy of a photo-excited electron-hole pair is likely to be converted to multi-excitons via carrier-multiplication. As a result, biexctions are known to play a crucial role for the intrinsic material gain (${g}_{m}$) in CsPbBr_3_ QDs^20^. However, the Auger process also shortens the decay time for the population inversion. In the case of colloidal CdSe QDs, the graded potential structure of multi-layered thick shells is used to suppress the Auger process, whereby electrically pumped ASE was successfully demonstrated^21^. Unfortunately, the Auger recombination time in CsPbBr_3_ QDs is known to be shorter than that in CdSe QDs^22^. Therefore, the gain is limited by the inherent nature of the perovskite material. CsPbBr_3_ nanosheets are expected to be less vulnerable to Auger processes due to their two-dimensional (2D) nature^23,24^, and the fast in-plane charge transport^25^ is also advantageous for electrically pumped lasers. So far, no gain study has been reported in CsPbBr_3_ nanosheets.

Alternatively, the optical confinement factor ($\gamma$) can be enhanced by waveguide geometry, plasmonic waveguides, and refractive index matching, resulting in an increase of net gain ($g=\gamma {g}_{{\rm{m}}}-\alpha$) despite the limits of small material gain (${g}_{m}$) and loss ($\alpha$). In the case of CsPbBr_3_ nanostructures, most interest is focused on material gain, and the waveguide effect was rarely studied^26,27^. As evidence of stimulated emission, an emergence of superlinear emission intensity growth and spectral narrowing are often shown with increasing excitation intensity^7–13^. However, a gain coefficient is necessary for a quantitative evaluation of the light amplification at a particular emission wavelength. A gain spectrum is also important to allow a determination of the energetic distribution of population inversion, whereby the microscopic origin of the gain can be revealed. When a gain is present, the ASE intensity shows an exponential increase as the propagation length increases, otherwise spontaneous emission gives rise to a linear increase with stripe length. Therefore, an optical gain coefficient at a particular wavelength can be obtained through the stripe length-dependent ASE intensity, which is known as the variable stripe length method (VSLM)^28^.

The VSLM is widely used for its simplicity, and most of the modal gain studies on MHPs have been based on the VSLM. However, various uncertainties can be involved when an optical gain coefficient is retrieved from the stripe length dependence of the ASE. These are very important to obtain a consistent gain coefficient, but usually overlooked. Therefore, possible pitfalls in both experimental implementation and numerical retrieval of $g$ should be considered^18,26,29^. For example, the intensity uniformity should be confirmed over the whole optical stripe, which is obtained from a Gaussian laser distribution using a cylindrical lens. An upper limit for the stripe length should be known for fitting due to the gain saturation. A threshold stripe length for amplification should also be considered when the stripe length is comparable to the stripe width.

In this work, we have studied the net gain of CsPbBr_3_ nanosheets in terms of temperature and excitation dependence, gain saturation, and the waveguide effect. We address possible pitfalls and limits of the old analysis in the VSLM; our stripe length-dependent gain coefficient $g(x)$ was estimated at a particular emission energy (ℏ*ω*), whereby we obtained a spectrum-length gain contour $g(\hslash \omega ,x)$. We found $g(\hslash \omega ,x)$ is quite useful in evaluating the gain saturation as a function of temperature and excitation intensity, whereby a representative average gain can be obtained. Additionally, we have also confirmed an enhancement of the modal gain in CsPbBr_3_ nanosheets through a micron scale waveguide structure, which was produced by a patterned substrate of polyurethane-acrylate (PUA).

## Results

**Fig. 1:**
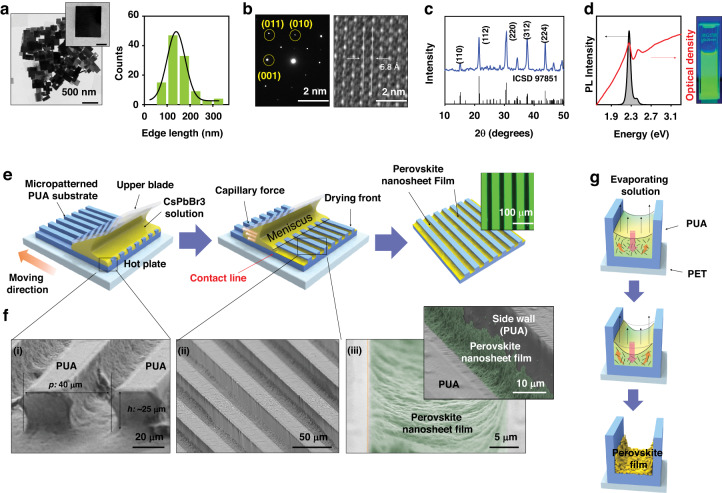
Characterization of CsPbBr_3_ nanosheets and the deposition process on a patterned PUA waveguide. **a** TEM image of two-dimensional CsPbBr_3_ nanosheets, where the inset shows a single-layered nanosheet with a scale bar of 100 nm, and the histogram shows the lateral size distribution. **b** SAED pattern and HRTEM image of CsPbBr_3_ nanosheets. **c** XRD pattern of CsPbBr_3_ nanosheets with the standard XRD pattern for the orthorhombic phase. **d** Absorption (optical density) and photoluminescence (PL) spectra of a green-emitting colloidal solution of CsPbBr_3_ nanosheets. **e** The deposition process to fill the patterned PUA substrate with CsPbBr_3_ nanosheets, an array of green fluorescence image of perovskite film is shown in the inset. **f** A set of SEM images shows the multi-stacked CsPbBr_3_ nanosheets on a patterned PUA substrate, where the front view (i), tilted side-view (ii), and magnified top-view (iii) of the sidewall at a selected single waveguide channel is shown. **g** Schematic illustration of the sequential deposition processes on a single microchannel

In Fig. 1a–f, the characterization of CsPbBr_3_ nanosheets and a schematic of the deposition process on a patterned PUA waveguide are shown, whereby a periodic array of perovskite stripes was obtained. As a first step, CsPbBr_3_ nanosheets were synthesized by a well-established hot injection method (see “Materials and methods”). Briefly, Cesium-oleate was used as a precursor, which accelerates the lateral growth of perovskite nanosheets with a high yield. To stabilize the colloidal solution of the two-dimensional (2D) nanosheets, oleic acid and oleylamine were used, where short carbon ligands (octanoic acid and octylamine) were combined to inhibit the uncontrolled vertical growth and stabilize the lateral growth mode in 2D nanosheets.

Figure 1a shows a transmission electron microscopy (TEM) image and a histogram of the lateral size distribution, where the inset TEM image clearly displays an isolated single CsPbBr_3_ nanosheet with a square shape, and we obtained the average lateral size to be (~140 ± 40 nm). To determine the crystallinity of the nanosheets, a selected area electron diffraction (SAED) pattern and a high-resolution TEM (HRTEM) image were measured. The SAED pattern (Fig. 1b) indicates an orthorhombic crystal phase (ICSD 97851, Pbnm (62), *a* = 8.207 $\mathring{\rm A}$, *b* = 8.255 $\mathring{\rm A}$, *c* = 11.759 $\mathring{\rm A}$), and the lattice fringe of 5.8 $\mathring{\rm A}$ was confirmed by the HRTEM image. XRD measurements were also conducted to determine the crystallinity of perovskite nanosheets (Fig. 1c). Based on the obtained diffraction patterns, the diffraction peaks were located at 15.16°, 21.46°, and 30.24°, which were matched with the (110), (112), and (220) crystal planes. It can be inferred that the perovskite nanosheets have an orthorhombic crystal structure, as the first three strong peaks, namely, (110), (112) and (220), together with the lattice fringe (5.8 $\mathring{\rm A}$)matched well with the standard orthorhombic perovskite structure^30^.

In Fig. 1d, absorption and PL spectra were measured with a CsPbBr_3_ nanosheet solution in a hexane solvent, which shows a green image under UV lamp excitation. Compared with the absorption peak at the lowest energy (~2.357 eV), the PL spectrum shows a narrow linewidth (~17 nm) without a low energy spectral tail. Therefore, the PL at room temperature can be attributed to 2D excitons confined in nanosheets with large lateral size (80 ~ 300 nm) and thin thickness (7–15 nm), when the exciton Bohr radius (*a*_x_ ~3.6 nm) and the binding energy (${E}_{x}^{b}$ ~ 40 meV) of bulk CsPbBr_3_ are considered^31^. Near the ground state absorption peak of CsPbBr_3_ nanostructures, two fine peaks have been shown to exist with a splitting energy of ~60 meV due to the electron-heavy hole (HH) and electron-light hole (LH) states^32^. In our case, they are too broad to be resolved from the absorption peak at ~2.357 eV. On the other hand, both absorption and PL spectra show a secondary peak near ~2.54 eV, which is separated by ~183 meV from the lowest peak. This can be explained by a bi-modal thickness distribution^33^; recent work reported that colloidal single crystal CsPbBr_3_ perovskite nanosheets with large lateral sizes (up to a few µm) and thickness of a few unit cells size (*<*5 nm) can be synthesized by introducing short and long ligands. When the volumetric ratio of short to long ligands was 0.67 in the perovskite solution, the mean lateral size of large nanosheets was ~5.16 µm. In the PL spectrum of the large nanosheets, two distinct peaks at 458 nm and 491 nm were also observed, which can be attributed to a bi-modal thickness distribution.

Figure 1e shows the sequential deposition process on a patterned structure, where the periodic microchannels were filled with a solution of CsPbBr_3_ nanosheets. Because the substrate consists of patterned PUA on polyethylene terephthalate (PET) film, the trapped CsPbBr_3_ nanosheets were stacked only on the trench of the microstructure, partially wetting on the sidewall of PUA, except the mesa areas. Therefore, light propagation along the channels can be guided, and the lateral light propagation can be amplified due to the enhanced optical confinement. The detailed photo- and nanoimprint-lithography for the micropatterned waveguides are explained in the Supplementary Information. We used capillary-directed self-assembly by employing a motorized translation stage (i.e., programmable moving substrate)^34^, where an inclined upper blade traps and sweeps the receding meniscus of the colloidal solution across the micropatterned substrate. The meniscus-assisted solution deposition process is known to yield a high coverage over a large area. Since we used a micropatterned structure instead of a flat substrate, the engraved channels confined the CsPbBr_3_ nanosheet solution. As shown in the middle panel of Fig. 1e, the trapped meniscus of the nanosheet solution quickly flows through the microfluidic channels, which is driven by capillary force and repetitive physical deposition. We found that the production yield of a uniformly stacked film of CsPbBr_3_ nanosheets was sensitive to the number of the deposition processes and the surrounding temperature as it is associated with the evaporation rate of the solution. Therefore, the temperature was carefully fixed at 60 °C for constant growth of CsPbBr_3_ nanosheets since the boiling point of the solvent (i.e., anhydrous hexane) is 69 °C and the nonvolatile solute (i.e., CsPbBr_3_ nanosheets) undergoes an unwanted phase transition. For complete evaporation of the solvent and simultaneous annealing, the patterned substrate (PUA/PET) was placed on a hot plate in a pre-heated state at 60 °C. After repetitive deposition processes (20 times) with multiple supplies of the nanosheet solution, an array of the nanosheet stripes separated by the PUA walls was obtained on a large scale (4 × 4 cm^2^). The right panel inset of Fig. 1e shows a clear fluorescence image of the CsPbBr_3_ nanosheet stripes. Therefore, light amplification is expected along each stripe efficiently.

Figure 1f shows scanning electron microscope (SEM) images of the micropatterned PUA structure, and the cross section in Fig. 1f(i) shows a 1:1 aspect ratio. Given the periodic micropatterned channels (40 µm) in Fig. 1f(ii), a conformal coating of CsPbBr_3_ nanosheets was successfully obtained only on the engraved trench areas as shown in Fig. 1f(iii). A highly magnified SEM image in Fig. 1f(iii) indicates that the closely packed CsPbBr_3_ nanosheets partially wet the surface of the PUA side walls by a conformally stacked layer. Therefore, our rapid assembly strategy is highly advantageous for producing spatially arranged multi-stacked perovskite nanosheets for a micro-laser array. It is also noteworthy that the generation of a micropatterned perovskite film is highly reproducible with a help of conventional lithographic techniques and capillary-directed self-assembly using simple organic solvent evaporation.

Figure 1g shows the sequential deposition process for the CsPbBr_3_ nanosheets on a single microchannel. When an aqueous CsPbBr_3_ nanosheets suspension was deposited, the volatile hexane solvent transported the nonvolatile solute of CsPbBr_3_ nanosheets into the trench area in a strictly controlled geometry by the fixed upper glass blade and the moving substrate. At this moment, all the solutes can be deposited and effectively trapped only on the trench area by the favorable surface wetting along with the lateral sliding of the meniscus, facilitating a highly diluted solution concentration level (C ~ 0.5 µg mL^−1^) (Supplementary Information). The consequent receding contact line is swept by a physical dragging force on the mesa surface and spontaneous solvent evaporation in the microfluidic channels, where the hydrodynamic flows and evaporative flux strictly drive the nanosheets into the trench area (middle panel in Fig. 1g)^35^. Given the largely reduced meniscus over the patterned substrate, the liquid thin film is continuously filled, and the nonvolatile solutes can be positioned in the deep grooved area (i.e., trench). On the other hand, the multiple supplies of the solution prohibit discontinuous patterned film formation by the coffee ring effect^36^. We intentionally applied a highly diluted solution in this process to optimize the thickness level of the nanosheets. With this effective guidance of the micropatterned substrate, the nanosheets were continuously deposited at the receding contact line at the air-liquid-solid interface, thereby anchored at each narrow microfluidic channel at the final stage of the complete solvent evaporation (the lower panel in Fig. 1g).

**Fig. 2:**
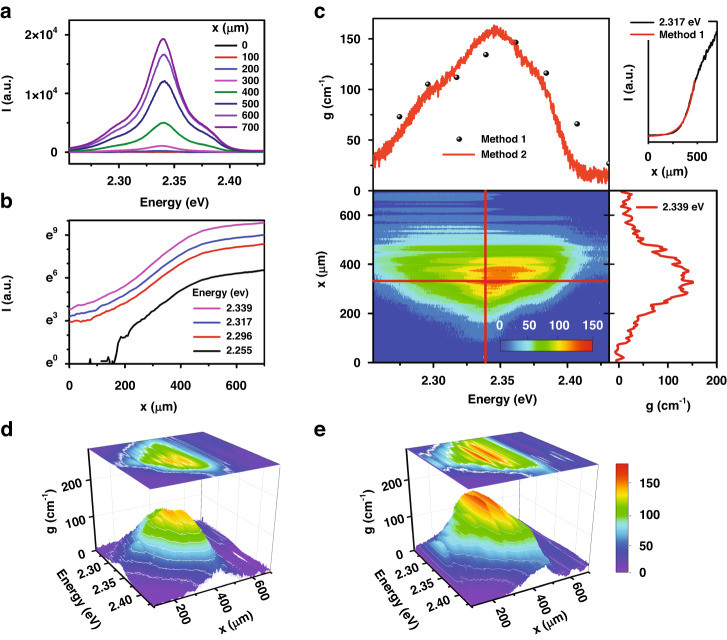
Optical net gain of CsPbBr_3_ nanosheets with and without a PUA waveguide at 4.2 K. **a** With increasing stripe length, ASE spectra of CsPbBr_3_ nanosheets were obtained without a PUA waveguide. **b** Stripe length dependence of the ASE intensity was plotted at various emission energies. **c** Eq. (4) (method 2) provides a contour of the net gain $g(\hslash \omega ,x)$ in the plane of spectral energy and stripe length under 0.07 µJcm^−2^ excitation. Inset shows the fitting of Eq. (2) (method 1). **d**, **e** $g(\hslash \omega ,x)$ of CsPbBr_3_ nanosheets without/with a PUA waveguide under 0.14 µJcm^−2^ excitation is shown, respectively

In Fig. 2, we show the net gain of CsPbBr_3_ nanosheets with and without a PUA waveguide using the VSLM (Supplementary Information). Figure 2a shows the ASE spectra of CsPbBr_3_ nanosheets on PET without a PUA waveguide with increasing stripe length. Whilst the ASE propagates along an excited stripe towards the sample edge, the ASE intensity $I(\hslash \omega ,x)$ becomes amplified by a net gain coefficient $g$, which consists of an optical confinement factor $\gamma$, an intrinsic material gain ${g}_{m}$, and a propagation loss *α*, i.e., $g=\gamma {g}_{{\rm{m}}}-\alpha$. Additionally, spontaneous emission is also involved with a spontaneous emission density ${J}_{{\rm{sp}}}({{\hslash }}\omega )$ and solid angle $\Omega$. Therefore, the stripe length dependence of the ASE intensity is given by:

1 $$\frac{{dI}\left({\hslash }\omega ,x\right)}{{dx}}=g\left({\hslash }\omega \right)I\left({\hslash }\omega ,x\right)+{J}_{{\rm{sp}}}\left({{\hslash }}\omega \right)\Omega$$

When stripe lengths are short, the gain coefficient is assumed to be independent to stripe length. Therefore, the solution to Eq. (1) is given by:

2 $$I\left({\hslash }\omega ,x\right)=\frac{{J}_{{\rm{sp}}}\left({\hslash }\omega \right)\Omega }{g\left({\hslash }\omega \right)}\left({e}^{g\left({\hslash }\omega \right)x}-1\right)$$

In Fig. 2b, the ASE intensity with stripe length $I(x)$ at various emission energies $\hslash \omega$ is shown. As the measured $I(x)$ shows an exponential increase $\sim {e}^{{gx}}$ up to ≲500 µm, a net gain coefficient can be obtained by fitting. For example, the ASE intensity selected at 2.317 eV (inset of Fig. 2c) fits well with Eq. (2) up to ~500 µm, giving rise to a gain coefficient of 112 cm^−1^. Alternatively, the two ASE intensities at $x$ and $2x$ can be used instead of fitting. Suppose Eq. (2) is still valid up to $2x$, the gain coefficient at a selected emission energy ${\hslash }\omega$ can be calculated by the ratio $I(2x)$ to $I(x)$ as:

3 $$g\left({\hslash }\omega \right)=\frac{1}{x}{\mathrm{ln}}\left(\frac{I\left({\hslash }\omega ,2x\right)}{I\left({\hslash }\omega ,x\right)}-1\right)$$

When compared with the fitting method of Eq. (2), which needs a number of tedious iteration processes to obtain optimum parameters, the calculation method using Eq. (3) is very convenient. However, uncertainties are unavoidable in both the fitting (Eq. (2)) and calculation (Eq. (3)) although these methods are widely used to obtain gain. As the stripe length increases, gain saturation becomes significant^26^. As a result, the ASE intensity $I(x)$ shows a considerable deviation from exponential growth, and the slope decreases. Therefore, a saturation length ${x}_{s}$ should be considered to apply to both Eq. (2) and (3), but the criterion for ${x}_{s}$ is quite ambiguous, as it also depends on emission energy. Therefore, the spectral dependence of ${x}_{{\rm{s}}}$ can modify the retrieved gain spectrum.

Additionally, the one-dimension approximation is no longer valid when the stripe length becomes comparable to the stripe width (∼50 µm). In this case, the amplification is inefficient ($g \sim 0$), and the ASE intensity shows a linear increase with stripe length $I\left(x\right) \sim {J}_{{\rm{sp}}}\Omega x$ up to a threshold length (${x}_{{\rm{th}}}$). It is also difficult to define the position of $x=0$ due to laser diffraction, whereby an offset signal ${I}_{{\rm{off}}}$ is observed. Therefore, Eq. (2) needs to be modified with ${x}_{{\rm{th}}}$ and ${I}_{{\rm{off}}}$, i.e., $I\left(x\right)=\frac{{J}_{{\rm{sp}}}\Omega }{g}\left(\exp \left[g\left(x-{x}_{{\rm{th}}}\right)\right]-1\right)+{I}_{{\rm{off}}}$, and the fitting begins at ${x}_{{\rm{th}}}$. The empirical fitting function is not a solution of the fundamental Eq. (1), and those limits are usually overlooked. Until now, most of the gain spectra reported for perovskite materials^7–13,18,19,27^ were based on either Eq. (2) or Eq. (3) (method 1).

When gain saturation is considered, intensity-dependent gain $g(I)$ is usually discussed. However, it is important to notice that the stripe length dependence is associated with stripe length dependence in the VSLM, i.e., $g\left(I(x)\right)=g(x)$. Specifically, the gain saturation is related to a non-uniform distribution of photons and carriers along the optical stripe^37^. Even if a uniform optical stripe is prepared by using a beam expander from the Gaussian intensity distribution of the laser beam^18^, the carriers become depleted as the propagating ASE is amplified. At the same time, a carrier diffusion also occurs toward the depleted region. Therefore, the interplay between photons and carriers gives rise to a non-uniform carrier distribution along the stripe, and gain saturation is unavoidable in the VSLM as the stripe length increases^18,26,29,37^. Although gain saturation is often observed in the VSLM, most of them were based on the intensity dependence. Those models are not intuitive nor convenient in data analysis^37^.

Based on the fundamental differential Eq. (2), we obtained the modal gain coefficient to be:

4 $$g\left({\hslash }\omega ,x\right)=\frac{\frac{{dI}\left({\hslash }\omega ,x\right)}{{dx}}-{J}_{{\rm{sp}}}\Omega }{I\left({\hslash }\omega ,x\right)}$$

where $\frac{{dI}}{{dx}}$ needs to be calculated from *I*(*x*), and the linear slope near ${x \,<\, x}_{\rm{th}}$ is used for ${J}_{{\rm{sp}}}\Omega$. In Fig. 2c, the gain in the CsPbBr_3_ nanosheets at 4.2 K is plotted against emission energy and stripe length $g(\hslash \omega ,x)$, which looks like a gain island floating on a sea level of $g=0$. On the upper side of Fig. 2c, the gain spectrum at $x=330$ µm is compared with that obtained by fitting Eq. (2). Whilst the well-known analysis (method 1) provides an average gain within 500 µm, our analysis of Eq. (4) (method 2) shows a gradual gain spectrum change with stripe length. On the right side of Fig. 2c, the stripe length dependence of the gain coefficient at 2.339 eV is also plotted. Note that the VSLM provides a net gain ($g=\gamma {g}_{m}-\alpha$). Most of the reported work on gain in perovskite materials prepared the samples by a drop-casting method. In this case, the optical confinement factor ($\gamma$) along the optical stripe is small. Compared with $g(\hslash \omega ,x)$ of CsPbBr_3_ nanosheets dropped on a PET substrate in Fig. 2d, e confirms that the PUA waveguide gives rise to a significant gain enhancement. Not only the gain coefficient but also the lateral gain area in the energy ($\hslash \omega$)-length ($x$) plane increases. This can be attributed to an increase of optical confinement factor ($\gamma$), and we have also confirmed this through a simplified simulation (Supplementary Information).

**Fig. 3:**
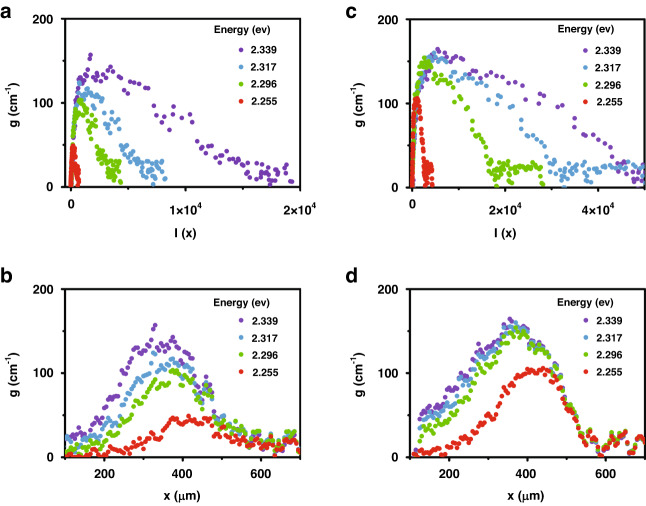
Gain saturation for ASE intensity *I*(*x*) and stripe length *x*. Under 0.07 µJcm^−2^ excitation, gain coefficients at various emission energies are plotted against ASE intensity and stripe length at 4.2 K with **a**, **b** and without **c**, **d** a waveguide structure, respectively

Our method with (4) is useful to study the gain saturation. In the case of inhomogeneous amplifiers, gain saturation is given $g\left(I\right)={g}_{0}/\sqrt{1+I/{I}_{{\rm{sat}}}}$, where the gain decreases from ${g}_{0}$ to ${g}_{0}/\sqrt{2}$ with the saturation intensity ${I}_{{\rm{sat}}}$. If the intensity dependence of the gain $g\left(I\right)$ is combined with Eq. (1), a stripe length dependence of the gain $g(x)$ can be obtained. However, experimental results are different from the theoretical predictions. Figure 3a shows $g\left(I\right)$ obtained from CsPbBr_3_ nanosheets dropped on a PET substrate at various emission energies. Because the ASE intensity $I\left(x\right)$ was measured with increasing $x$, length-dependent gain $g(x)$ can also be obtained (Fig. 3b), i.e., $g\left(I(x)\right)=g(x)$. Obviously, the gain decrease for large *I* do not fit with ${({1+I/{I}_{\rm{sat}}})}^{-0.5}$, nor is the gain maximized with ${g}_{0}$ for small $I(x)\cong 0$. The small gain at short stripe lengths (${x \,<\, x}_{{\rm{th}}}$) can be attributed to the limit of the one-dimensional model. Unless the optical stripe length is long enough compared with the stripe width, sufficient amplification is not achieved. In Fig. 3c, d, the enhanced gain coefficients with the PUA waveguide are plotted for increasing ASE intensity $g\left(I\right)$ and stripe length $g(x)$, respectively. These results show the optical confinement enhancement depends on the spectral energy. On the other hand, the gradual gain decrease beyond 450 µm is associated with carrier depletion, associated with the interplay between carriers and photons^29^. Whilst the photons are amplified toward the stripe edge, the carriers become depleted along the stripe. This also leads to a transient variation of the carriers and photons. Even if the excitation beam is uniform along the stripe, the carrier density becomes non-uniform. This effect becomes significant for *>* 450 µm giving rise to a gain saturation.

It is known that biexcitons play an essential role in the origins of intrinsic gain in CsPbBr_3_ nanostructures^8,38^. Nevertheless, the gain lifetime of biexcitons becomes shortened as Auger recombination becomes dominant, and the biexciton Auger lifetime in CsPbBr_3_ QDs is an order of magnitude shorter than that in colloidal CdSe QDs^22^. Surprisingly, it was found that the Auger process becomes suppressed as the confinement dimension increases from QDs (0D), nanorods (1D), and nanoplates (2D)^23,24^. In the case of 2D CsPbBr_3_ nanoplatelets (NPLs), the Auger lifetime was known to be proportional to the lateral area^23^. Therefore, CsPbBr_3_ nanosheets are expected to be less vulnerable to Auger effects. As the lateral size (*L* = 80 ∼300 nm) of our CsPbBr_3_ nanosheets is far larger than the exciton Bohr radius (*a*_x_ ~ 3.6 nm), a 2D free carrier model can be a possible candidate to explain the gain^11^; as the free carrier density increases in a 2D structure, the effective chemical potential increases over the band edge, determining the upper limit of the gain spectrum. The gain spectrum below the band edge also broadens as a result of various many-body effects such as carrier scattering, biexcitons, and bandgap renormalization.

However, AFM images of the CsPbBr_3_ nanosheets (Supplementary Information) show that the thickness of a single CsPbBr_3_ nanosheet is not constant (7–15 nm). Because the vertical thickness is far smaller than the lateral size, the confinement energy levels are dominated by the thickness. Therefore, the thickness variation of a single nanosheet gives rise to a valley-like potential energy structure, and excitons are likely to be localized near the thickness maximum. The effective localized area might be similar to that of CsPbBr_3_ NPLs, where the area varies from 350–1100 nm^2^. In this case, a 2D center-of-mass exciton confinement model can be considered^39^, where the confinement levels are determined by a whole exciton instead of separate electrons and holes. As a result, the stochastic thickness variation possibly results in a broad spectral range with a different density of the states.

We should also note that our excitation fluence is smaller by two orders of magnitude than the reported threshold fluence for stimulated emission at room temperature. This difference can be attributed to the large repetition rate (80 MHz) of our laser pulses (140 fs) and the low temperature (4.2 K). Because the time interval between laser pulses (~12.5 ns) corresponding to 80 MHz is shorter than emission lifetime, the residual carriers can accumulate in a similar manner to quasi-continuous excitation. We found the threshold fluence at 4.2 K is smaller by one order of magnitude compared to that at room temperature (~2 µJcm^−2^)^40^.

**Fig. 4:**
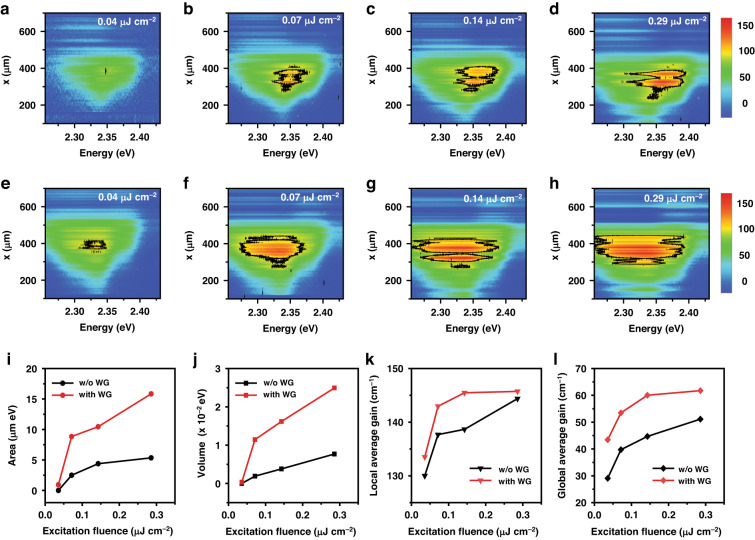
Excitation dependence of the net gain at 4.2 K. **a**–**d** For increasing excitation fluence, $g(\hslash \omega ,x)$ for CsPbBr_3_ nanosheets is plotted, where the black contour line corresponds to $g=130$ cm^*−*1^. **e**–**h** For CsPbBr_3_ nanosheets with a PUA waveguide, the excitation fluence dependence of $g(\hslash \omega ,x)$ becomes enhanced significantly. **i**, **j** As the excitation fluence increases, we obtain the area and the volume for local conditions of $g\,\ge \,130$ cm^*−*1^ for the CsPbBr_3_ nanosheets with and without the waveguide, respectively. **k** Excitation fluence dependence of the local average gain for $g\,\ge \,130$ cm^*−*1^ obtained from (**i**, **j**). **l** Excitation fluence dependence of the global gain average for $g\,\ge\, 0$ cm^-1^

We have also investigated the excitation dependence of the gain at 4.2 K through a spectrum-length contour plot $g(\hslash \omega ,x)$. In Fig. 4a–d, the $g(\hslash \omega ,x)$ of the CsPbBr_3_ nanosheets on a PET substrate is shown as a function of increasing excitation fluence up to 0.29 µJ/cm^2^. In Fig. 4e–h, the $g(\hslash \omega ,x)$ of the CsPbBr_3_ nanosheets with a PUA waveguide is shown again as a function of fluence. Compared with the $g(\hslash \omega ,x)$ of CsPbBr_3_ nanosheets without a PUA waveguide (Fig. 4a–d), both the vertical gain magnitude and the lateral energy-length area for the $g\ge 130$ cm^*−*1^ show a significant increase due to the increased optical confinement factor. In addition, the gain contour shows periodic patterns along length. Although gain oscillations were observed in a noise level (Fig. 3d), the periodic feature in the large gain range becomes significant for increasing excitation. This result is possibly associated with mode propagation in the waveguide structure (Supplementary Information).

As the excitation grows, the gain spectrum usually broadens. When gain saturation becomes involved in the VSLM, the available stripe length for $g \,> \,0$ also needs to be analyzed. We have introduced an area in the energy-length plane. For example, considering the reference contour line of $g=130$ cm^*−*1^, which is the maximum gain coefficient for our CsPbBr_3_ nanosheets under 0.04 µJ/cm^2^ excitation, the area of $g\ge 130$ cm^*−*1^ in the energy-length plane can be obtained ($\iint d\hslash \omega {dx}$). In Fig. 4i, the excitation dependence of the local area for the reference $g\,\ge \,130$ cm^*−*1^ is plotted. For CsPbBr_3_ nanosheets without a PUA waveguide, the local area shows a logarithmic increase with excitation fluence. With a PUA waveguide, both the spectral width and the available length for $g\ge 130$ cm^*−*1^ increase, resulting in a significant increase in the local area.

Furthermore, we calculated the local volume $\iint g(\hslash \omega ,x)d\hslash \omega {dx}$, where the two-dimensional gain is integrated within the area for $g\ge 130$ cm^*−*1^. In Fig. 4j, the calculated local volume of CsPbBr_3_ nanosheets is shown for increasing excitation fluence. Whilst the increase of the local area saturates (Fig. 4i), the local volume shows a linear increase with excitation fluence. With a PUA waveguide, the local volume increases. Given this area and volume, we can calculate the local average gain. As shown in Fig. 4k, the excitation dependence of the local average gain for CsPbBr_3_ nanosheets with and without a PUA waveguide are compared. This analysis might be useful to evaluate a gain medium when a cut-off gain threshold is present for lasing operation.

Similarly, a global ($g\ge 0$ cm^*−*1^) gain average can also be obtained, where the whole area and volume are included for $g\ge 0$ cm^*−*1^. In Fig. 4l, the global average gain for CsPbBr_3_ nanosheets with and without a PUA waveguide are compared for increasing excitation fluence, where both show a similar logarithmic increase. In the case of a 2D gain medium, the excitation dependence of the gain is known to be $\sim {g}_{0}(1+\mathrm{ln}(I/{I}_{{\rm{th}}}))$, where the gain con- verges to ${g}_{0}$ near the threshold excitation power *I*_th_^41^. Therefore, Fig. 4l supports a 2D model. In the presence of a PUA waveguide, the global gain also shows a significant enhancement compared to the local gain. Whilst PL is associated with the relaxation and transfer of excited carrier population, gain is governed by the density of states, whereby the population inversion density is determined. It was known that the PL spectrum of CsPbBr_3_ nanostructures is dominated by the defect states of Br vacancies and surface states, and the energy levels located below and above the exciton state were estimated by the thermal activation energy^42–45^. Together with the crystal disorder, the spatial inhomogeneity of nanosheet thickness also results in localized states (Supplementary Information). Consequently, the gain of CsPbBr_3_ nanostructures is expected to appear with a large spectrum width (~20 meV). The defect states are distributed spatially in a stochastic manner and strongly localized. Unless stripe length is long enough, consecutive amplification of the localized states is inhibited by the absorption loss in the stripe. Likewise, the gain of the localized states develops as excitation increases. Those features are seen in the excitation dependence of $g(\hslash \omega ,x)$ at 4.2 K (Fig. 4); the gain spectrum width becomes increased with increasing stripe length up to ~450 µm, resulting in an area increase in the energy-length plane.

The gain of the localized states is also sensitive to temperature, but temperature-dependent gain is reported rarely. Most of the temperature dependence in CsPbBr_3_ nanostructures was reported in terms of the PL spectrum. Because the valence band maximum of CsPbBr_3_ is determined by the hybridization between the 4*p* orbital of Br and the 6 *s* orbital of Pb, lattice expansion reduces the hybridization between the two orbitals, resulting in a bandgap increase with temperature^46^. Additionally, localized defect states become activated, and the carrier-phonon interactions becomes enhanced. Consequently, the gain deteriorates with a spectral shift as the temperature increases.

Despite the localized states of crystal disorder and thickness inhomogeneity, perovskite materials were known to show extreme flexibility, whereby the so-called dynamic disorder occurs in a picosecond time scale^47^. As a result, the diffusion lengths of free carriers and excitons become extended up to several micrometers. This explains the efficient transport of perovskites despite defects and localized states. Therefore, the two-dimensional center-of-mass confined excitons and the localized states coexist in the broad spectrum of perovskite nanosheets (Fig. S11 in Supplementary Information).

**Fig. 5:**
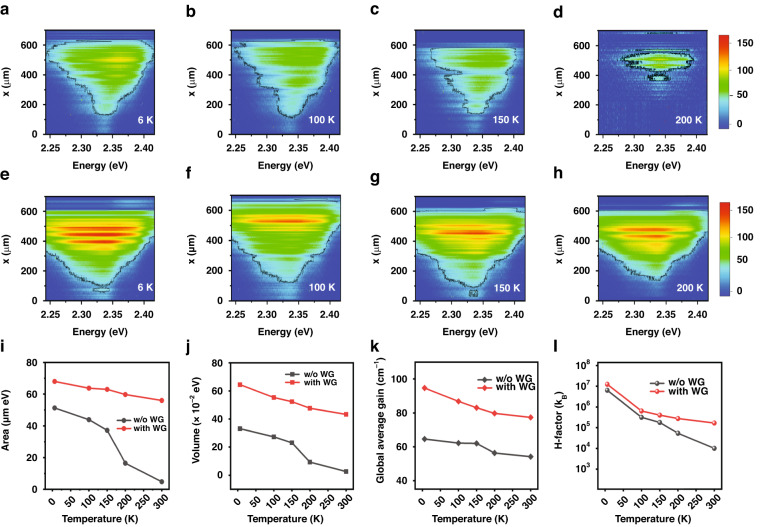
Temperature dependence of net gain under 0.14 µJ/cm^2^ excitation. **a**–**d** For increasing temperature, $g(\hslash \omega ,x)$ of CsPbBr_3_ nanosheets on PET substrate are plotted, where the black contour line corresponds to $g=30$ cm^*−*1^. **e**–**h** For CsPbBr_3_ nanosheets with PUA waveguide, thermal stability of $g(\hslash \omega ,x)$ becomes enhanced significantly. **i**, **j** For increased temperature, the area and the volume for the global condition of $g \sim 0$ cm^*−*1^ are obtained for CsPbBr_3_ nanosheets with and without the waveguide, respectively. **k** Temperature dependence of the global average gain was obtained from (**i**, **j**). **l** Temperature dependence of H-factor as a measure of thermal gain stability, where room temperature (300 K) results were also used (Fig. S18 in Supplementary)

Our gain contour map $g(\hslash \omega ,x)$ is useful for evaluating thermal stability. In Fig. 5a–d, the $g(\hslash \omega ,x)$ of the CsPbBr_3_ nanosheets on a PET substrate is shown for increasing temperature from 6 K up to 200 K, which is comparable with the LO-phonon energy of CsPbBr_3_ (19 meV)^43^. The $g(\hslash \omega ,x)$ of the CsPbBr_3_ nanosheets with a PUA waveguide (Fig. 5e–h) was also obtained for comparison. Given the global gain condition of $g \sim 0$ cm^*−*1^, the area (Fig. 5i) and the volume (Fig. 5j) were obtained in the energy-length plane for increasing temperature. In Fig. 5k, temperature dependence of the global average gains was compared with and without a PUA waveguide, respectively.

To quantify thermal gain stability, H-factor was also obtained by devising the global gain volume by temperature. In Fig. 5l, temperature dependence of the H-factor is shown in units of the Boltzmann constant (k_B_). Gain deteriorates with increased temperature but decrease of the H-factor is relatively suppressed when the waveguide structure is used. With a PUA waveguide, we found that not only the gain is enhanced but also the temperature stability improves significantly^44,45^. A possible reason would be increased thermal conduction via the PUA waveguide. It is noticeable that the channel width (~20 µm), where CsPbBr_3_ nanosheets are stacked, and the wall width (~20 µm) of the PUA waveguide were designed intentionally with respect to the width of an optical stripe length (~50 µm). When an optical stripe is located at the optimum position, only a single channel is excited within the two side walls. Therefore, an efficient heat dissipation may occur along the wall, whereby thermal gain quenching becomes suppressed.

## Discussion

We have found that a patterned waveguide structure enhances both gain and thermal stability due to the increased optical confinement and heat dissipation. By excitation and temperature dependence of gain contour $g(\hslash \omega ,x)$ in the energy-length plane, the gain origins in CsPbBr_3_ nanosheets were attributed to the 2D center-of-mass confined excitons and the localized states arising from the inhomogeneity of thickness and the defect states.

## Materials and methods

### Materials

Lead bromide (PbBr_2_, 99.999% trace metals basis), cesium carbonate (Cs_2_CO_3_, reagentPlus, 99%), 1-octadecene (ODE, technical grade, 90%), oleylamine (OLA, 70%), oleic acid (OA, 90%), octanoic acid (OctAc, 99%), octylamine (OctAm, 99%), hexane (anhydrous, 95%), and Molecular sieves, 4 ^˚^A were purchased from Sigma-Aldrich. PUA resin was supplied by Min- uta Technology. AZ 9260 was obtained from AZ electronic materials. AZ 300 MIF was purchased from Merck. Sylgard 184 was supplied by Dow Corning. ODE, OA and OLA were dehydrated using molecular sieves before use, other chemicals were used as received without further purification.

### Preparation of Cs-oleate solution

The Cs-oleate solution was prepared by the method reported by Dou et al.^48^, Cs_2_CO_3_ (0.16 g) and OA (10 ml) were loaded into 25 ml 3-neck flask and dried under vacuum at 120 °C for 50 min, and then heated under N_2_ to 140 °C until all Cs_2_CO_3_ reacted with OA. Cs-oleate solution was pre-heated to 100 °C before injection.

### Synthesis of CsPbBr_3_ nanosheets

ODE (10 ml), PbBr_2_ (0.069 g), OA (0.3 ml), OLA (0.3 ml), OctAc (0.5 ml), and OctAm (0.5 ml) were loaded into 50 ml 3-neck flask and dried under vacuum for 20 min at 100 °C. After complete solubilization of the PbBr_2_, the temperature was raised to 150 °C under N_2_. In total, 0.8 ml of Cs-oleate solution was swiftly injected. After 20 min, the reaction was stopped by using an ice-water bath to room temperature. The crude solution was dispersed in anhydrous hexane and centrifuged at 2000 rpm for 5 min. The supernatant was discarded, and the precipitate was re-dispersed in anhydrous hexane. This isolation process was repeated again, and the supernatant was collected separately for further process.

### Preparation of line-patterned PUA substrate

In order to prepare the PDMS mold, a drop of photoresist (PR) was placed on a chrome-coated glass substrate (100 × 100 × 0.5 mm), a spin-coating process was performed at a spinning speed of 750 rpm for 30 s. The coated substrate was then soft-baked at the temperature of 110 °C for 10 min. UV radiation with 1800 mJcm^−2^ illuminated the substrate placed under a patterned mask, the development was processed using a developer solution for 20 min. Rinsed with DI water, the patterned mold was completely dried on a hot plate. To fabricate a PDMS replica mold, a PDMS prepolymer with a curing agent solution was poured on the patterned mold and cured at 80 °C for 1 h. Subsequently, an appropriate amount of PUA resin was dropped on the PDMS mold to create a line-patterned substrate. Then, a PET guide film was located on the PUA resin and uniformly pressed using a roller for conformal contact between the PUA resin and the PET film. The PUA resin was then exposed to UV light (*λ* = 365 nm). After fully curing, the PDMS mold was carefully detached from patterned PUA substrate.

### Capillary-directed self-assembly of perovskite nanosheets

The line-patterned PUA substrate (2 × 2 cm^2^) was used without an additional cleaning process. The deposition process for the CsPbBr_3_ nanosheets on the line-patterned substrate was conducted on a hot plate set at 60 °C to facilitate the evaporation of the volatile solvent (i.e., hexane) and the deposition. Next, an upper blade was installed at a 30° angle, and the colloidal perovskite solution was injected in a confined geometry containing the upper blade and the lower substrate. A trapped meniscus was formed by capillary force in this restricted geometry, and 20 cycles of one-way motion were performed at a speed of 2.5 mm*/*s by a computer-controlled translation stage. A uniformly stacked perovskite nanosheet film layer on PUA microfluidic channels was obtained through the repetitive capillary-directed self-assembly process. After the deposition process, the multi-stacked perovskite nanosheets on a patterned substrate were stored in a vacuum desiccator for further measurement and experiments.

### Characterization of CsPbBr_3_ nanosheets and line-patterned substrate

The synthesized CsPbBr_3_ nanosheets were observed by TEM (TALOS, F200X) under an acceleration voltage of 200 kV to confirm their morphologies. HRTEM and SAED analyses also were performed on transmission electron microscope (TALOS, F200X). The thickness of the CsPbBr_3_ nanosheets was measured by an AFM (Park systems, NX10). The UV-Vis absorption spectra were measured on an Evolution 220 UV-Visible Spectrophotometer. The PL spectra were recorded by a Fluorescence Spectrophotometer (Hitachi, F-7000). The multi-stacked perovskite nanosheets on the patterned PUA substrate were observed by SEM (Gemini, SUPRA 40 VP) and Digital Microscope (CELENA®S Digital Microscope). For optical gain measurement, frequency-doubled 400 nm pulses (140 fs duration) were used, which are operating at 80 MHz repetition rate, and the fundamental pulses of 800 nm are pumped by 18 W continuous laser.

### Supplementary information

Supplementary information for Gain enhancement of perovskite nanosheets by a patterned waveguide: excitation and temperature dependence of gain saturation

## Data Availability

The data that support these findings are available from the corresponding author upon request. Source data of all figures are provided with this paper.
