# Supplementary material for: Gain enhancement of perovskite nanosheets by a patterned waveguide: excitation and temperature dependence of gain saturation
Source: Light Sci Appl. 2023 Nov 24;12:285. doi: 10.1038/s41377-023-01313-0 (PMC10673887; doi:10.1038/s41377-023-01313-0)
Supplement: Supplementary file 1 — Supplementary information for Gain enhancement of perovskite nanosheets by a patterned waveguide: excitation and temperature dependence of gain saturation [file 41377_2023_1313_MOESM1_ESM.pdf]

Supplementary information for Gain enhancement of perovskite nanosheets by a patterned waveguide: excitation and temperature dependence of gain saturation

Inhong Kim[1], Ga Eul Choi[1], Ming Mei[1], Minwoo Kim[1], Minju Kim[1], Young Woo Kwon[2], Tae-In Jeong[1], Seungchul Kim[1], Suck Won Hong[1], Kwangseuk Kyhm[1], and Robert A. Taylor[3]

[1]Department of Opto & Cogno Mechatronics Engineering, RCDAMP, Pusan National University, Busan 46241, Republic of Korea
[2]Department of Nano-Fusion Technology, Pusan National University, Busan 46241, Republic of Korea
[3]Clarendon Laboratory, Department of Physics, University of Oxford, Oxford, OX1 3PU, U.K

Capillary-directed self-assembly of perovskite nanosheets

In order to prepare the PDMS mold, a drop of photoresist was placed on a chrome-coated glass substrate (100 × 100 × 0.5 mm), a spin-coating process was performed at a spin speed of 750 rpm for 30 s. The coated substrate was then soft-baked at a temperature of 110°C for 10 minutes. The substrate was then placed under a patterned mask and illuminated by UV radiation at 1800 mJ/cm^2, the substrate and PR developing process took 20 min using developer solution. After rinsing with deionized water, the patterned mold was completely dried on a hot plate. To fabricate a PDMS replica, PDMS prepolymer with curing agent solution was poured on the patterned mold and cured at 80°C for 1 h. Subsequently, an appropriate amount of PUA resin was dropped on the PDMS mold to create a line-patterned substrate. Then, PET guide film was located on the PUA resin and rolled using a roller for conformal contact between the PUA resin and the PET film. The PUA resin was then exposed to UV light at $\lambda = 365$ nm. After curing completely, the PDMS mold was carefully detached from the patterned PUA substrate. Figure S1 shows the sequential

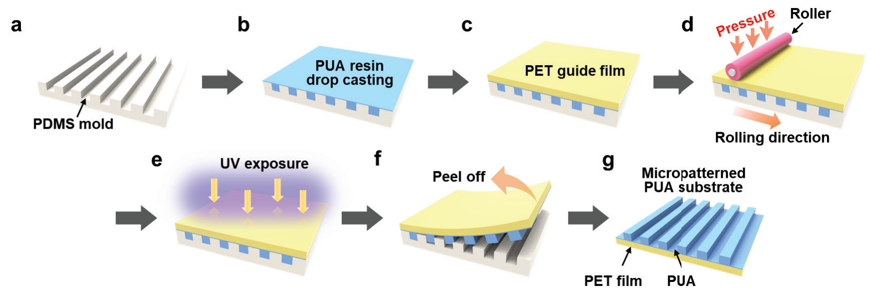

Figure S1: Schematic illustration of the sequential molding process to prepare a PUA substrate via nano-imprint lithography. **a** The PDMS master mold is prepared by conventional lithography (SU-8). **b** PUA resin was cast by a spin coating process. **c** A PET backing guide film was contacted on top of the PUA resin. **d** A roller was used with mild pressure to form a conformal wetting between the PET backing and the PUA resin. **e** UV exposure (365 nm) was applied through the PET film (i.e., step-and-flash imprint lithography). **f-g** Peeling off the patterned PUA/PET film.

molding process used to prepare a PUA substrate via nano-imprint lithography.

Patterned PUA waveguide fabrication

The line-patterned PUA substrate ($2 \times 2\,cm^2$) was used without any additional cleaning process. The deposition process for the $CsPbBr_3$ nanosheets on the line-patterned substrate was conducted on a hot plate set at 60°C to facilitate the evaporation of the solvent (hexane) and the deposition. Next, the upper blade was installed at a 30°angle, and the colloidal perovskite solution was injected in a confined geometry containing the upper blade and the lower substrate. The trapped meniscus was formed by capillary force in this restricted geometry, and 20 cycles of one-way motion were performed at a speed of $2.5\,mms^{-1}$ by a computer-controlled translation stage. A uniformly stacked perovskite nanosheet film layer on PUA microfluidic channels was obtained through this repetitive capillary-directed self-assembly process. After the deposition process, the multi-stacked perovskite nanosheets on a patterned substrate were stored in a vacuum desiccator for further measurement and experiments. Figure S2 show an SEM image of the uniformly stacked $CsPbBr_3$ nanosheets on a line-patterned PUA substrate, which is fabricated by capillary directed self-assembly.

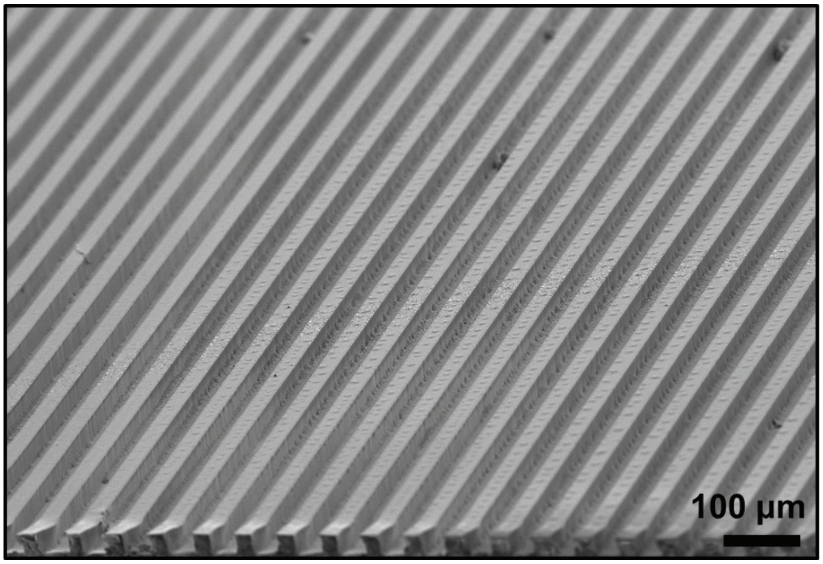

Figure S2: SEM image of uniformly stacked perovskite nanosheets on a line-patterned PUA substrate, which is fabricated by capillary-directed self-assembly.

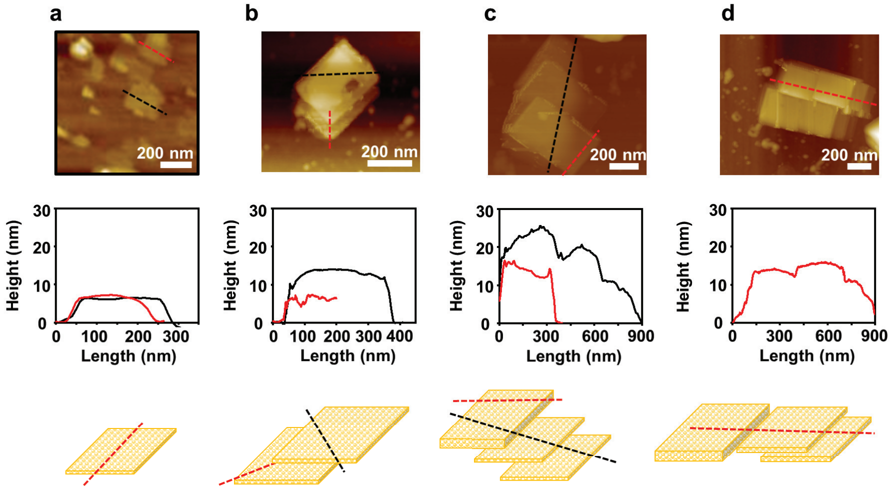

Figure S3: Given various AFM images of $CsPbBr_3$ nanosheets on a SiO_2/Si substrate, the height profiles were obtained along dotted line in red and black, respectively. **a** Thin ($\sim$ 7 nm) nanosheets. **b** A pair of thin nanosheet are stacked. **c** Thick ($\sim$ 15 nm) nanosheets on thin nanosheets. **d** Stacked thin nanosheets are located near a thick nanosheet.

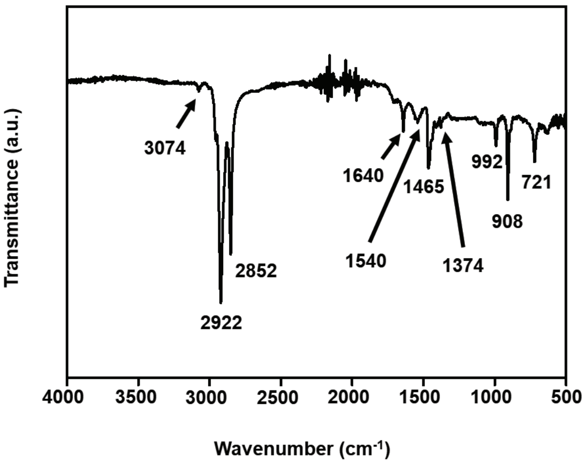

Figure S4: Fourier transform infrared (FTIR) spectra of $CsPbBr_3$ nanosheets.

AFM and FTIR

The thickness of the $CsPbBr_3$ nanosheets was measured using an AFM (Park systems, NX10). In Figure S3, various AFM images of $CsPbBr_3$ nanosheets on a SiO_2/Si substrate are shown, where height profiles were obtained. According to our AFM analysis, two different thickness groups were found. Thin (~ 7 nm) nanosheets can be stacked but the presence of thick (~ 15 nm) nanosheets was observed as shown schematically.
Fourier transform infrared (FTIR) spectra measurements on $CsPbBr_3$ nanosheets was performed, where several characteristic peaks can be observed with the presence of specific functional groups. The peak located at 3074 cm^{-1} revealed C-H stretching vibrations. In addition, asymmetric and symmetric stretching vibrations of the C-H single bond at 2922, 2852, and 1374 cm^{-1} were also detected, and C-H single bond bending in the $-CH_2-$ group was observed at 1465 and 721 cm^{-1}. C=O bond corresponding to the -COO- group of OA appeared at 1540 cm^{-1}, and $\equiv$C-H bending vibrations were observed at 992 and 908 cm^{-1}. From these results, we found that some of the organic ligands that could not be removed during the cleaning process remained in the deposited $CsPbBr_3$ nanosheets in the channel.

Time-resolved PL of $CsPbBr_3$ nanosheets

Temperature dependent PL and time resolved PL measurements were performed. PL spectrum for $CsPbBr_3$ nanosheets in solution (fresh and 2-day-old)

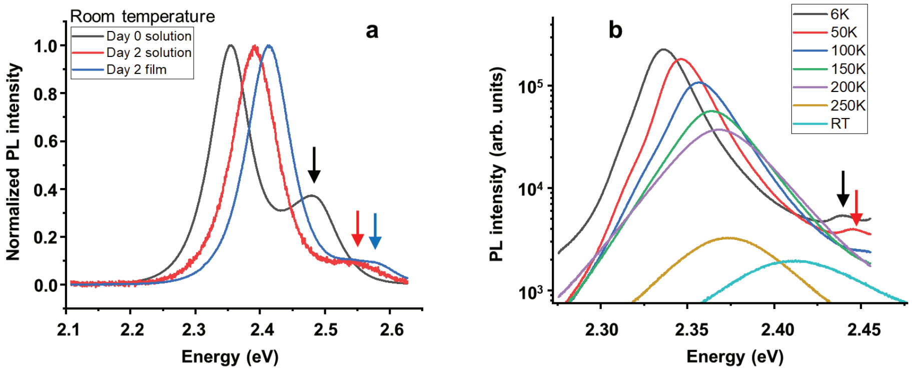

Figure S5: **a** Comparison of PL spectrum for $CsPbBr_3$ nanosheets in solution (fresh and 2-day-old) and film, which was made on the 2nd day. **b** Temperature dependent PL spectrum of the film measured on the 3rd day from 6 K up to room temperature.

and film, which was made on the 2nd day, were shown Figure S5. The secondary peak at high spectrum energy was significant when solution was fresh (0th day of production), but it became suppressed after a couple of days. When the 2-day-old solution became a film, an additional spectral shift was observed. Temperature dependent PL spectrum of the film measured on the 3rd day from 6 K up to room temperature was shown Figure S5 **b**. It was obvious that the secondary peak at high spectrum energy was suppressed with increasing temperature. As shown in Figure S6 and S7, time resolved PL (TRPL) spectrum of perovskite nanosheets solution and film at room temperature were obtained. The TRPL spectrum from solution sample shows a two-step decay. Fast (τ_f) and slow decay times (τ_s) were obtained at different spectrum energy, and the former is possibly associated with localization or trapping process.

Time-resolved PL of film sample was also measured at room temperature, and fast- and slow- decay times were compared over various spectrum energy. For decreased energy, the both decay times increase. While the secondary peak shows a significant two-step decay, the fast component increases gradually for decreased energy. Two-step decay of the primary peaks is not as significant as that of the secondary peak. This result suggests a carrier transfer from the strongly confined localized states to relatively low-lying energy states.

As shown in Figure S8, time-resolved PL at 6 K was also measured from the film, where fast (τ_f) and slow decay times (τ_s) were obtained at different spectrum energy. The decay times show a gradual increase with decreased spectrum energy, and the secondary peak near 2.44 eV is nearly suppressed. The fast decay times at 6K become shortened compared to those at room temperature as thermal activation becomes suppressed as temperature decreases. Nevertheles, the PL spectrum at 6 K is still broad, and this can be expained by height inho-

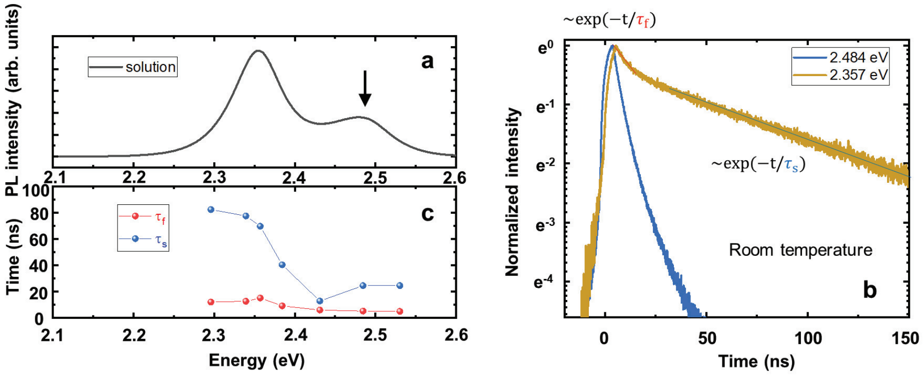

Figure S6: Time-resolved PL of $CsPbBr_3$ nanosheets in solution. **a** PL spectrum of $CsPbBr_3$ nanosheets in solution. **b** Comparison of the two time-resolved PL at the primary (2.357 eV) and secondary (2.484 eV) peak, where the two-step decay was characterized with fast (τ_f) and slow (τ_s) decay times. **c** Fast (τ_f) and slow (τ_s) decay times were plotted over various spectrum energy.

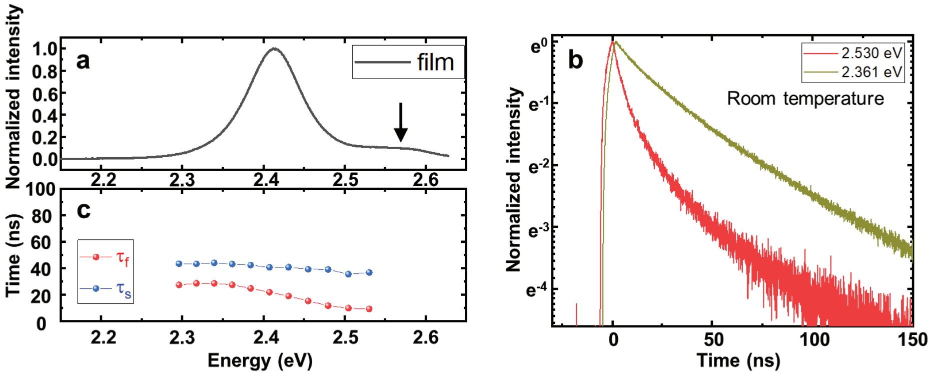

Figure S7: Time-resolved PL of $CsPbBr_3$ nanosheets in film was measurd at room temperature. **a** PL spectrum of $CsPbBr_3$ nanosheets in film (3-day-old). **b** Comparison of the two time-resolved PL at the primary (2.361 eV) and secondary (2.530 eV) peak, where the two-step decay was characterized with fast (τ_f) and slow (τ_s) decay times. **c** Fast (τ_f) and slow (τ_s) decay times were plotted over various spectrum energy.

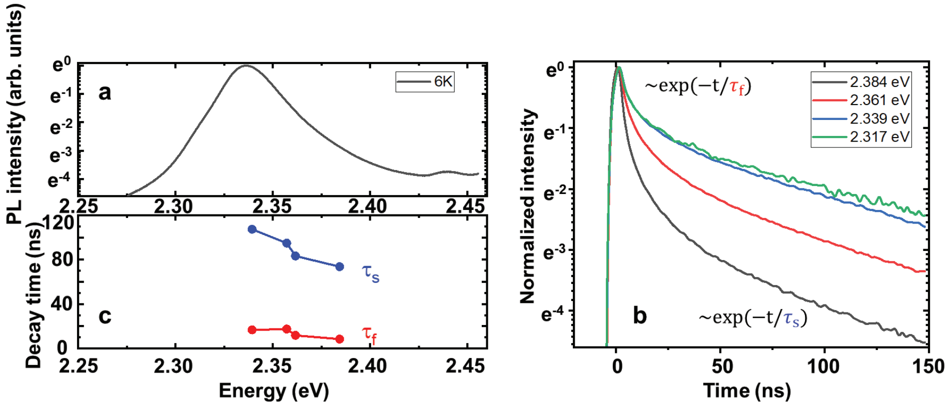

Figure S8: Time-resolved PL of $CsPbBr_3$ nanosheets in film was measurd at 6 K. **a** PL spectrum of $CsPbBr_3$ nanosheets in film (3-day-old). **b** Comparison of the two time-resolved PL at various spectrum energy, where the two-step decay was characterized with fast (τ_f) and slow (τ_s) decay times. **c** Fast (τ_f) and slow (τ_s) decay times were plotted over various spectrum energy.

mogeneity as well as crystal inhomogeneity, resulting in various localized states distributed over a wide spectrum range even at 6 K.

Time-resolved PL at the PL peak energy were compared for increasing temperature (Figure S9). Two-step decay at low temperature becomes a nearly monotonic decay at room temperature, and the dominant slow (τ_s) component decreases. The suppression of fast component can be explained by thermal excitation of the localized states.

As shown in Figure S10, we have monitored degradation process from fresh solution state. In 2-day-old solution sample, the suppression of secondary peak occurs. This can be attributed to aggregation and stacking of thin nanosheets. A film was made on the 2nd day, and the secondary peak becomes suppressed further on the 3rd day. On the 4th day, the film was exposed to picosecond pulsed laser (404 nm) for 10 hours, and PL intensity was decreased with 60% reduction. This can be attributed to inefficient heat dissipation within the film. Another film was exposed to air (maintained with 50% humidity and 20°C temperature). The 8-day-old film shows a decrease of decay time, where two-step decay process becomes significant compared to that of the 3-day-old film. This result suggest that sample degradation causes non-radiative trapped states.

As shown in Fig. S11 schematically, $CsPbBr_3$ nanosheets are associated with two kinds of disorders. One is crystal inhomogeneity such as the defect states of Br vacancies and surface states. Additionally, thickness inhomogeneity of nanosheets also causes localized states as the confinement energy is dominated by the vertical size. Consequently, emission is observed over a broad spectrum although non-radiative trap states are also present.

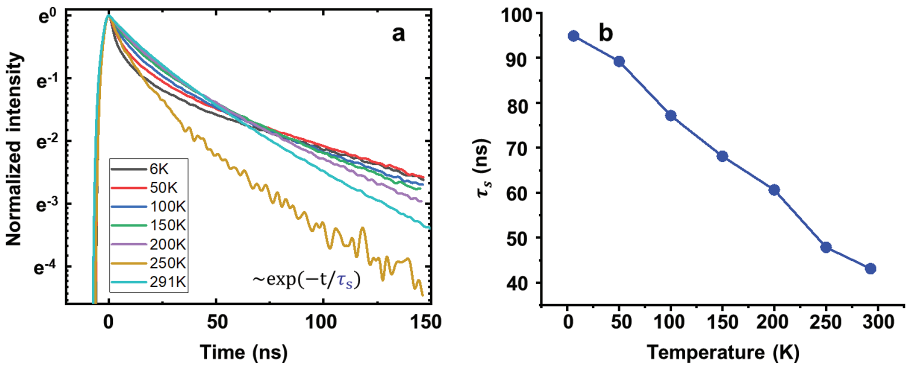

Figure S9: **a** For increasing temperature, time-resolved PL of $CsPbBr_3$ nanosheets in film was measured at PL peak energy. While two-step decay is significant at low temperatures, the fast decay time (τ_f) becomes increased gradually with increased temperature.**b** Temperature dependence of the slow decay time (τ_s).

Variable stripe length method

The optical arrangement for the VSLM setup is shown schematically in Figure S12**a**. For excitation, 400 nm laser pulses (1) were used, which were obtained via second harmonic generation from fundamental laser pulses at 800 nm from a mode-locked Ti:Sapphire laser operating at 80 MHz repetition rate. The circular laser spot becomes a stripe shape after passing through a cylindrical lens (2), and is focused on the sample. The optical stripe length (x) was adjusted by a movable laser beam block (3) with a $\Delta x = 5\,\mu$m step size. Because of the Gaussian intensity distribution of a laser spot, an inhomogeneous intensity still remains along the optical stripe (4), and the stripe has a vertical width (Δx). The intensity distribution along the stripe can be measured by the knife-edge method or a beam profiling instrument, and the uniformity can also be enhanced by using a beam expander before the cylindrical lens. Within the measurement stripe length range, we have confirmed that the stripe beam intensity shows 20 % decrease over a whole stripe length $\sim 700\,\mu$m.

The edge emission spectrum (5) was collected by a lens pair (6), and detected by charge-coupled device (7). Because the sample is mounted in a cryostat, the distance from the sample edge to the lens pair is far larger than the stripe length itself. Therefore, the solid angle can be assumed to be constant (i.e. $\Omega(x) \simeq \Omega$). Figure S5**b** shows how a circular laser beam becomes a rectangular stripe with an elliptical shape, where the length (Δx) and the width (Δy) of

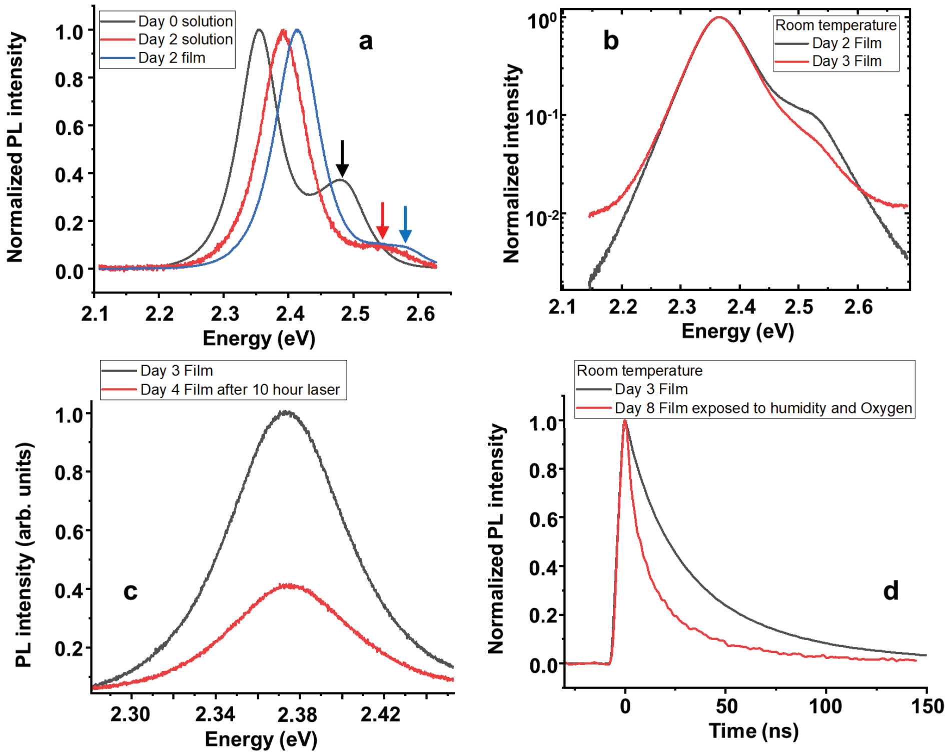

Figure S10: Degradation of $CsPbBr_3$ nanosheets in solution and film. **a** Comparison of PL spectrum for solution (fresh and 2-day-old) and film, which was made on the 2nd day. **b** PL spectrum of film on the 2nd and 3rd day, where the secondary peak near $\sim 3.56\,\mathrm{eV}$ becomes suppressed. **c** PL spectrum of 4-day-old film under 10 hour laser excitaion and 3-day-old film. **d** Time-resolved PL decay of 3-day-old film and 8-day-old film, which was exposed to humidity and oxygen.

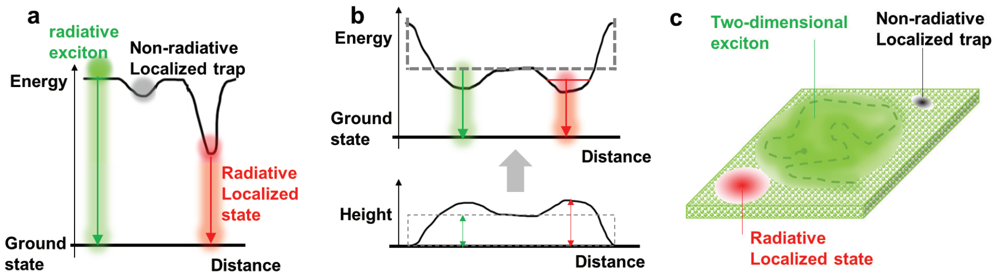

Figure S11: Optical transitions of perovskite nanosheets are explained schematically. **a** Excitons and localized states contribute to radiative process while localized trap states are involved in non-radiative process. **b** Thickness variation of perovskite nanosheets, gives rise to localized states. **c** While the thickness inhomogeneity gives rise to localized states, crystal disorder results in non-radiative trap states with defects.

a rectangular stripe can be determined by a pair of beam blocks. The Δx is changed by a motorized moving beam block with a $5\,\mu$m step whilst the width remains constant $\Delta y \sim 50\,\mu$m.

Analysis of the stripe length-dependent ASE intensity

When an optical stripe length x is set by the movable beam block, the amplified spontaneous emission (ASE) at x can be collected from the open edge, where the ASE intensity $I(\hbar\omega, x)$ depends on both the energy $(\hbar\omega)$ and stripe length (x). Additionally, spontaneous emission should be considered, which is given by $J_{\mathrm{sp}}\Omega$, where J_{sp} is the spontaneous emission density and Ω is the solid angle, respectively. Suppose the optical stripe is a one-dimensional amplifier, then the differential equation for $I(\hbar\omega, x)$ becomes:

$$\frac{\mathrm{d}I(\hbar\omega, x)}{\mathrm{d}x} = gI(\hbar\omega, x) + J_{\mathrm{sp}}(\hbar\omega)\Omega \tag{1}$$

Suppose the modal gain coefficient $g(\hbar\omega)$ at a certain energy $\hbar\omega$ is constant, the solution to Eq. (1) is then:

$$I(\hbar\omega, x) = \frac{J_{\mathrm{sp}}(\hbar\omega)\Omega}{g(\hbar\omega)}(\mathrm{e}^{g(\hbar\omega)x} - 1) \tag{2}$$

To obtain a modal gain coefficient $g(\hbar\omega)$, Eq. (2) is compared with the stripe length dependent ASE intensity selected at a particular emission energy $(\hbar\omega)$, where the two fitting parameters of $g(\hbar\omega)$ and $J_{\mathrm{sp}}(\hbar\omega)\Omega$ need to be optimized. However, unavoidable differences are always observed in the VSLM experiment.

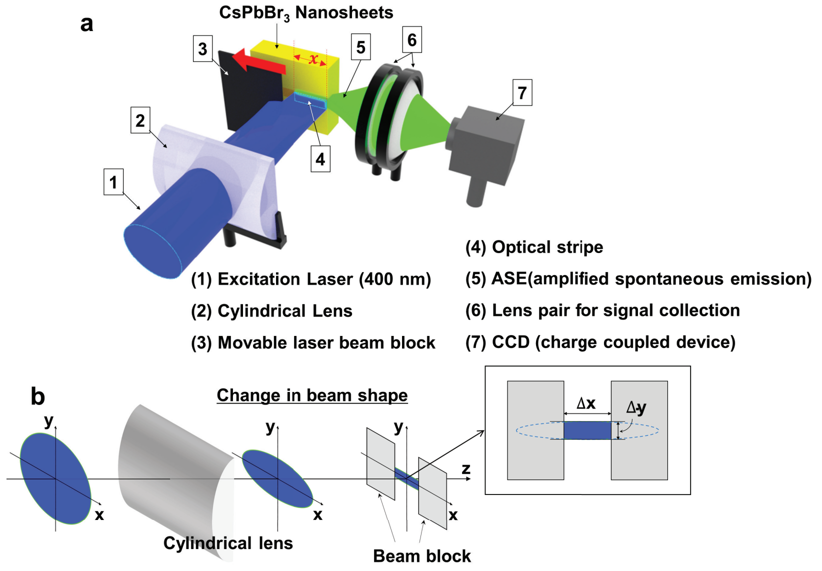

Figure S12: **a** A schematic of the vertical stripe length method (VSLM) setup. **b** A circular beam becomes a rectangular stripe by using a cylindrical lens.

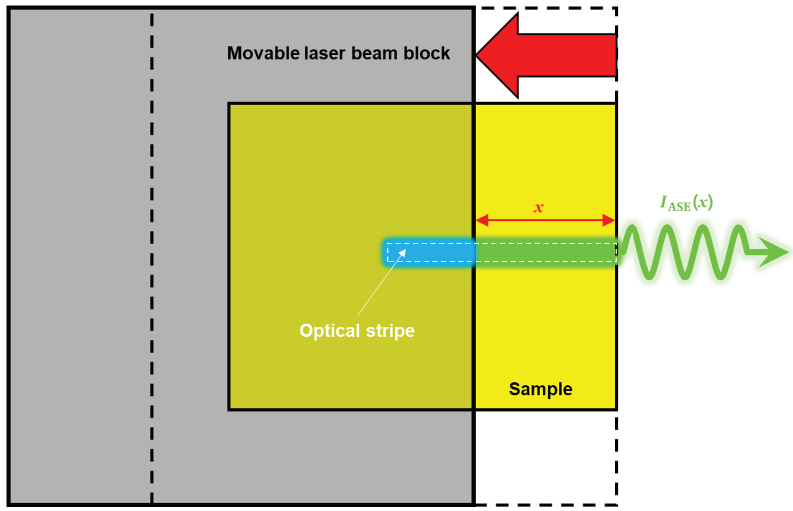

Figure S13: Schematic of one-dimensional amplification.

First, $I(\hbar\omega, x)$ shows a linearly increase when the stripe length x is less than a threshold value length x_{th} due to the finite stripe width. The stripe length needs to be longer than the stripe width of 50 μm. Second, an offset signal can be observed at the position of $x = 0$ due to laser diffraction. Therefore, Eq. (2) needs to be modified as:

$$I(x) = \frac{J_{\mathrm{sp}}\Omega}{g}\Big(\exp[g(x - x_{\mathrm{th}})] - 1\Big) + I_{\mathrm{off}} \tag{3}$$

Furthermore, the empirical fitting of function of Eq.(3) is not a solution of Eq.(1), and x_{th} and I_{off} depend on experimental conditions. For example, x_{th} increases for a small gain medium. Even if the same medium is used, x_{th} also depends on the emission energy. In the case of strongly localized defect states, which which occurs at low spectral energy, x_{th} is relatively long.

As our motivation is a systematic study on temperature dependence, film sample was mounted on a cold finger of cryostat. In this case, the edge emission diverges as it passes through cryostat window.

This simulation was also verified in experiment, where only a single waveguide was excited using a narrow width of an optical stripe (50 micron) as shown in Fig. S16-(b).

Third, an upper limit for the stripe length has to be selected, the so-called saturation length x_{sat} as $I(\hbar\omega, x)$ deviates gradually from exponential growth ($\sim \mathrm{e}^{g(\hbar\omega)x}$) near the saturation length x_{sat}. Consequently, the fitting has to be done within a selected range of stripe lengths ($x_{\mathrm{th}} < x < x_{\mathrm{sat}}$). The saturation length x_{sat} is also determined with uncertainties, and the emission energy dependence is often ignored. Consequently, the current fitting method provides an average gain over a particular range ($x_{\mathrm{th}} < x < x_{\mathrm{sat}}$), but uncertainties are unavoidable in the gain magnitude and spectrum width.
As an alternative method, the gain can be obtained from the fundamental differential equation (1) as

$$g(\hbar\omega, x) = \frac{\frac{\mathrm{d}I(\hbar\omega,x)}{\mathrm{d}x} - J_{\mathrm{sp}}\Omega}{I(\hbar\omega, x)} \tag{4}$$

For the first step, $\frac{\mathrm{d}I}{\mathrm{d}x}$ needs to be calculated from $I(x)$ at a selected $\hbar\omega$. For the second step, the constant linear slope near $x < x_{\mathrm{th}}$ is used for $J_{\mathrm{sp}}\Omega$. When the noise of $\frac{\mathrm{d}I}{\mathrm{d}x}$ is too large to define a constant slope, an average slope or the slope at a particular length can be used for consistency. For the third step, the numerator $\frac{\mathrm{d}I(\hbar\omega,x)}{\mathrm{d}x} - J_{\mathrm{sp}}\Omega$ is divided by the denominator $I(\hbar\omega, x)$. In this case, the small noise of $I(\hbar\omega, x)$ is likely to be amplified. Therefore, the offset near $x \simeq 0$ is useful in the last calculation, and noise filtering is necessary, otherwise the retrieved gain spectrum shows a small signal-to-noise ratio as the denominator noise becomes amplified. Nevertheless, the shape of gain spectrum is dominated by the derivative spectrum $\frac{\mathrm{d}I(\hbar\omega,x)}{\mathrm{d}x}$, and it is also useful to evaluate the degree of amplification. As a detail of the data calculation, the length differentiation to a data matrix $I(\hbar\omega, x)$ needs to be performed first at a selected $\hbar\omega$, and the derivative spectrum $\frac{\mathrm{d}I(\hbar\omega,x)}{\mathrm{d}x}$ can then be obtained by transposing the resultant

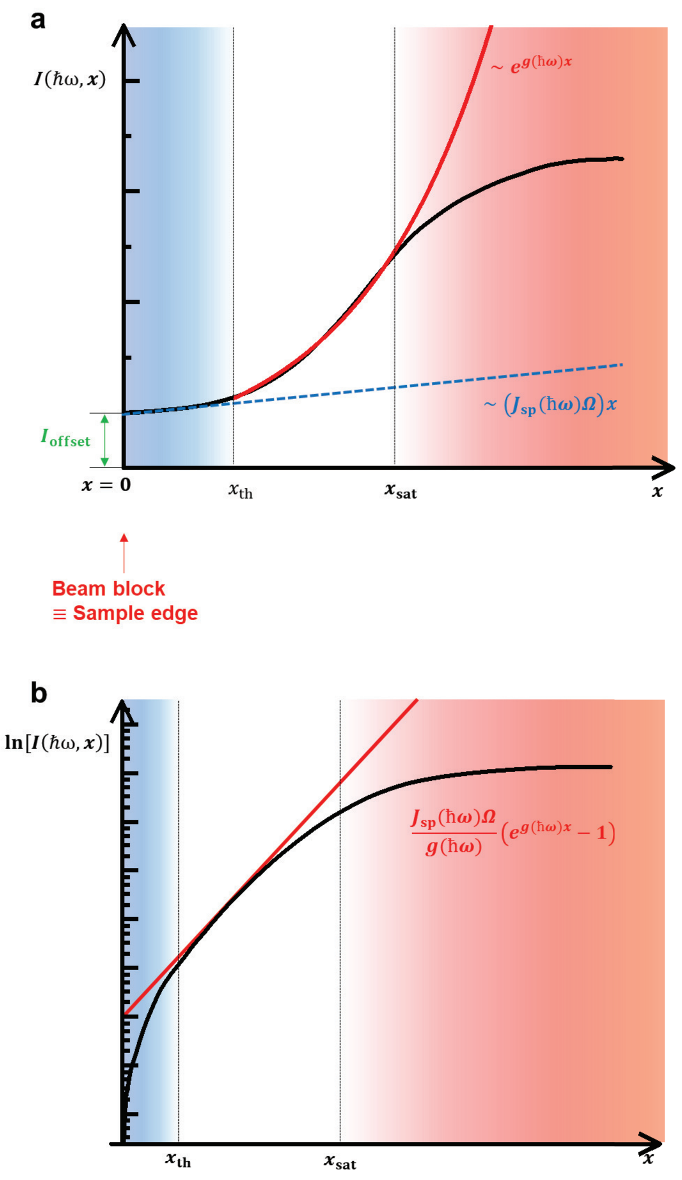

Figure S14: $I(\hbar\omega, x)$ and $J_{\text{sp}}(\hbar\omega)\Omega$ at **a** linear and **b** logarithmic scales.

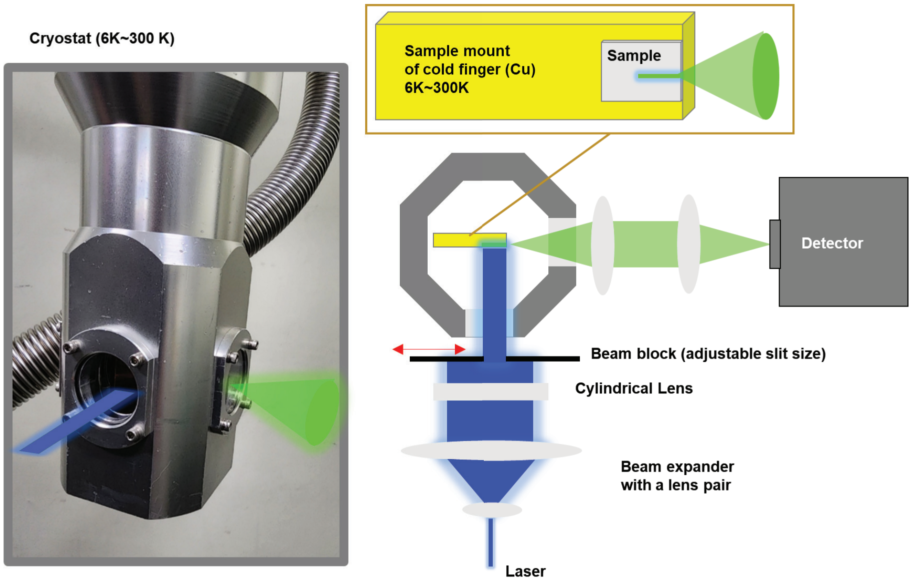

Figure S15: Schematic of $CsPbBr_3$ NSs film mounted in a cryostat for temperature-dependent optical gain measurement.

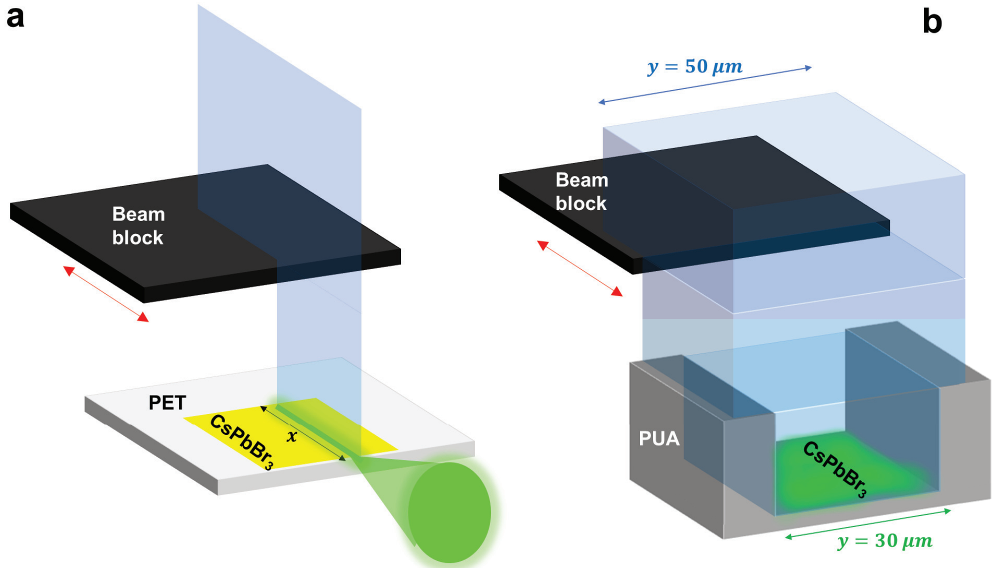

Figure S16: Schematic illustration of $CsPbBr_3$ nanosheets without **a** and with **b** PUA waveguide after movable beam block.

matrix.

Theoretical simulation for beam propagation within a waveguide

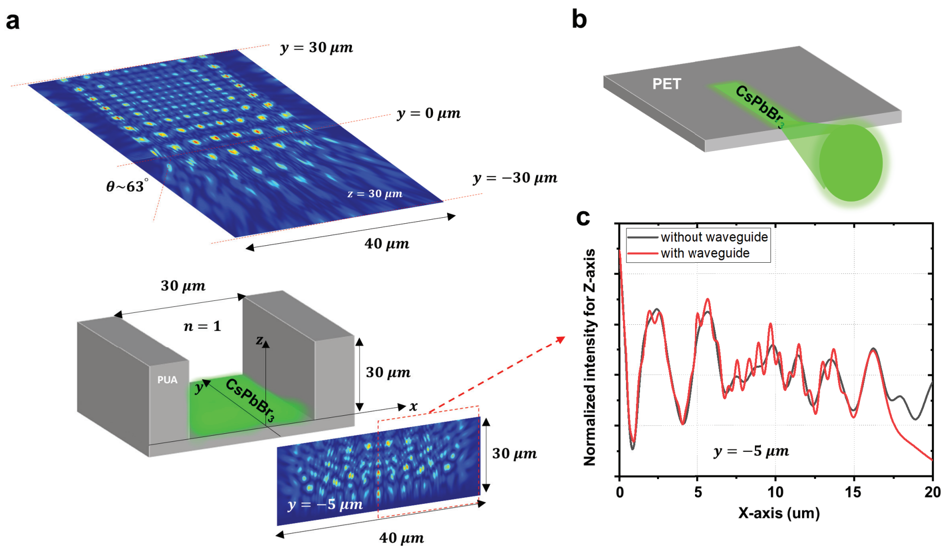

Figure S17: Theoretical simulation of optical propagation along the rectangular waveguide with 30 μm width and 30 μm height.

A finite-difference time-domain (FDTD) simulation was calculated to confirm the optical confinement effect. For intuitive understanding of the optical confinement, the point dipole sources were aligned along the wavefront across the waveguide to mimic the Huygens principle, where the refractive indices of CsPbBr3 ($n \sim 2$) and PUA/PET ($n \sim 1.5$) were used for the dominant ASE emission wavelength.

Fig. S17-(a) shows how the propagation modes are guided by the PUA wall. Although the xy plane selected at $z = 30\,\mu$m is located on an open area of the waveguide structure, guided mode patterns are still seen. The simulation also shows that the edge emission diverges with an angle of 63 degree to the exit xz plane ($y = 0$). Compared with the edge emission of CsPbBr3 film on PET substrate without a patterned waveguide (Fig. S17-(b)), the guiding effect is significant as shown in Fig. S17-(c). Although the xz plane is separated with $5\,\mu$m (i.e. $y = -5\,\mu$m) from the exit xz plane ($y = 0$), the edge still shows a waveguiding effect still affects. This simulation supports the presence of optical confinement in the PUA structure, whereby an enhancement of net gain can be

achieved.

Spectrum-length gain contour at room temperature

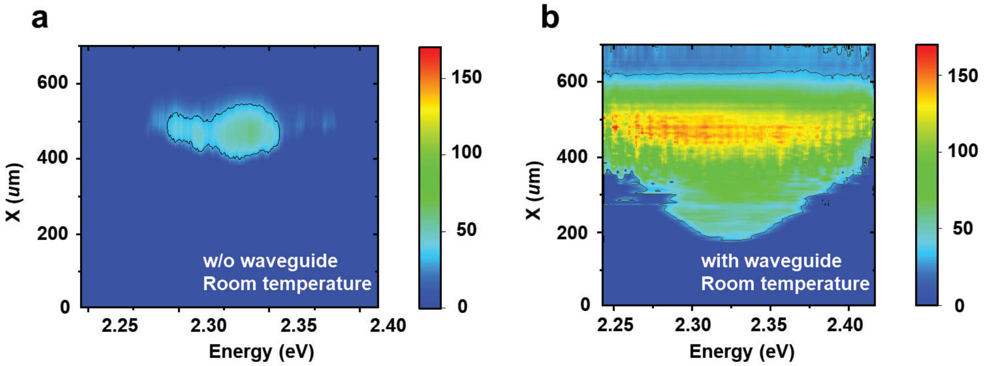

Figure S18: The spectrum-length gain contour of $CsPbBr_3$ nanosheets at room temperature were compared without **a** and with a PUA patterned waveguide **b**.

Sample stability

To date, $CsPbBr_3$ perovskite has been the subject of extensive research due to its excellent optoelectronic properties. However, it is known to suffer from poor stability issues, particularly when exposed to light, heat, water, and oxygen (Nature Materials, 17, 394-405 (2018)). These environmental factors can lead to degradation and phase transitions in $CsPbBr_3$ perovskite (The journal of Physical Chemistry C, 127, 24 (2023)), which adversely affect its performance and long-term reliability. $CsPbBr_3$ perovskite nanosheets can be easily degraded by light, heat, moisture, and oxygen. We stored the colloidal solution samples either in a freezer at -20°C and at room temperature, then the samples were evaluated by a series of measurements such as PL, absorbance, and TEM. In the case of PL, the peak of the sample stored at -20°C showed a tendency to maintain the integrity of its initial PL property, while the TEM image did not show any significant difference (Figure S19).

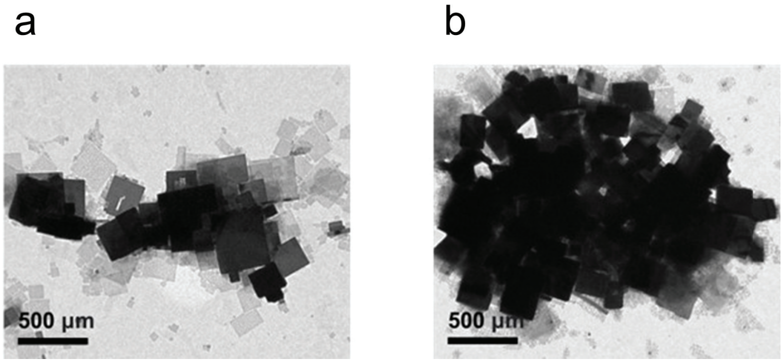

Figure S19: TEM images of the $CsPbBr_3$ perovskite nanosheets, in the case of the sample stored at -20°C **a** and ambient condition **b**.
